# Crystal structure of di-μ-hydroxido-bis{[*N*,*N*′-bis­(2,6-di­methyl­phen­yl)pentane-2,4-diiminato(1–)]zinc}

**DOI:** 10.1107/S160053681401736X

**Published:** 2014-08-06

**Authors:** Joshua A. Goodner, Brandon J. Powers, Douglas R. Powell, Lei Yang

**Affiliations:** a Department of Chemistry, University of Central Arkansas, 201 Donaghey Avenue, Conway, AR 72035, USA; b Department of Chemistry and Biochemistry, University of Oklahoma, Norman, OK 73019, USA

**Keywords:** crystal structure, zinc, hydroxide bridging ligand, β-diketiminate ligand

## Abstract

The title compound, [Zn_2_(C_21_H_25_N_2_)_2_(OH)_2_], is a binuclear zinc complex formed by two bidentate β-diketiminate (nacnac) ligands and two μ-hydroxide O atoms, bridging two mononuclear units into a centrosymmetric dimeric unit. Each Zn^2+^ cation is coordinated by two N-donor atoms from the nacnac ligand and two O-donor atoms of hydroxide anions to give a distorted tetra­hedral coordination environment. The Zn—O bond lengths are 1.9643 (13) and 2.0022 (14) Å, and the two Zn—N bond lengths are 1.9696 (14) and 1.9823 (14) Å. The distance between the two Zn^2+^ cations in the dimer is 2.9420 (4) Å. Although hydroxide groups are present in the complex, no classical hydrogen-bonding inter­ations are observed because of the bulky β-diketiminate ligands.

## Related literature   

For similar compounds with a [Zn_2_(OH)_2_] diamond core structure supported by β-diketiminate ligands, see: Cheng *et al.* (2001 ▶); Chisholm *et al.* (2002 ▶); Gondzik *et al.* (2014 ▶); Schulz *et al.* (2011 ▶). For the geometry index of four-coordinated metal cations, see: Yang *et al.* (2007 ▶).
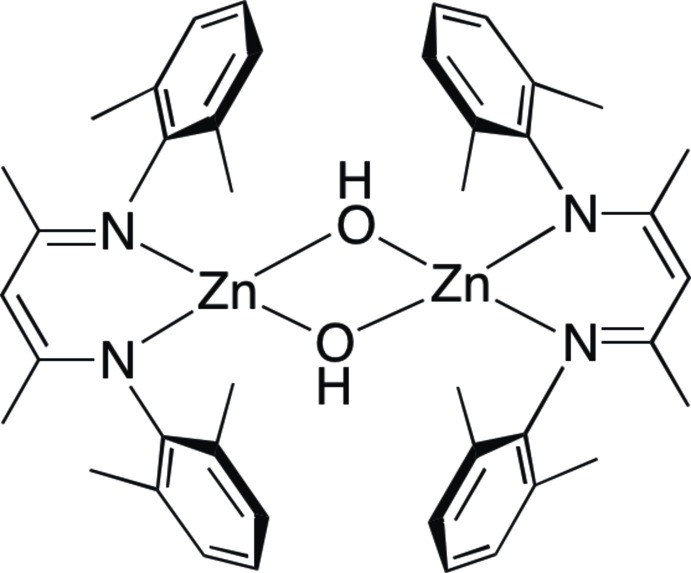


## Experimental   

### Crystal data  

[Zn_2_(C_21_H_25_N_2_)_2_(OH)_2_]
*M* *_r_* = 775.61
Monoclinic, 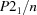

*a* = 12.8736 (4) Å
*b* = 8.7682 (3) Å
*c* = 17.4530 (5) Å
β = 105.222 (2)°
*V* = 1900.95 (10) Å^3^
*Z* = 2
Mo *K*α radiation
μ = 1.30 mm^−1^
*T* = 100 K
0.46 × 0.23 × 0.23 mm

### Data collection  

Bruker APEX CCD diffractometer
Absorption correction: multi-scan (*SADABS*; Bruker, 2007 ▶) *T*_min_ = 0.586, *T*_max_ = 0.754
22627 measured reflections
3730 independent reflections
3576 reflections with *I* > 2σ(*I*)
*R*_int_ = 0.016

### Refinement  

*R*[*F* ^2^ > 2σ(*F* ^2^)] = 0.029
*wR*(*F* ^2^) = 0.079
*S* = 0.99
3730 reflections
230 parameters
H atoms treated by a mixture of independent and constrained refinement
Δρ_max_ = 1.06 e Å^−3^
Δρ_min_ = −0.25 e Å^−3^

### 

Data collection: *SMART* (Bruker, 2007 ▶); cell refinement: *SAINT* (Bruker, 2007 ▶); data reduction: *SAINT*; program(s) used to solve structure: *SHELXTL* (Sheldrick, 2008 ▶); program(s) used to refine structure: *SHELXL2012* (Sheldrick, 2008 ▶); molecular graphics: *SHELXTL*; software used to prepare material for publication: *SHELXL2012* (Sheldrick, 2008 ▶).

## Supplementary Material

Crystal structure: contains datablock(s) I, New_Global_Publ_Block. DOI: 10.1107/S160053681401736X/wm5037sup1.cif

Supporting information file. DOI: 10.1107/S160053681401736X/wm5037Isup2.docx

Structure factors: contains datablock(s) I. DOI: 10.1107/S160053681401736X/wm5037Isup3.hkl

Supporting information file. DOI: 10.1107/S160053681401736X/wm5037Isup5.cdx

**Fig. 1.**
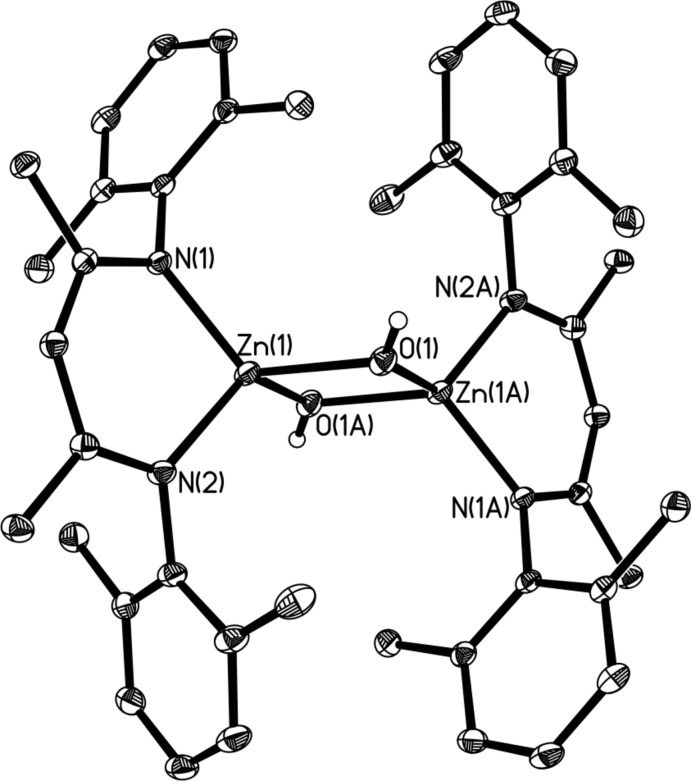
The molecular structure of the title complex. Anisotropic displacement ellipsoids were drawn at the 30% probability level. Hydrogen atoms, except for the two oxygen donor atoms, have been omitted for clarity. [Symmetry code A: -x+2, -y+2, -z+2.]

. DOI: 10.1107/S160053681401736X/wm5037fig1.tif
The mol­ecular structure of the title complex. Anisotropic displacement ellipsoids were drawn at the 30% probability level. Hydrogen atoms, except for the two oxygen donor atoms, have been omitted for clarity. [Symmetry code A: −x+2, −y+2, −z+2.]

CCDC reference: 1013307

Additional supporting information:  crystallographic information; 3D view; checkCIF report

## supplementary crystallographic information

### S1. Synthesis and crystallization

The complex was synthesized from the reaction of water with the [Zn(Et)(*N*,*N*'-bis­(2,6-di­methyl­phenyl)­pentane-2,4-diiminato)] complex in Et_2_O. The solvent was removed under vacuum after the reaction, and a white powder was collected. Recrystallization of the white powder in saturated Et_2_O at 253 K led to the formation of colorless crystals.

### S2. Refinement

The positions of hydrogen atoms bonded to carbon atoms were initially determined by geometrical considerations and refined by using a riding model. The hydrogen atom bonded to the oxygen atom was located in a difference map, and its position was refined independently. Hydrogen atom displacement parameters were set to 1.2*U*_eq_ (1.5 for methyl) of the respective parent atom.

### Crystal data

**Table:**

[Zn_2_(C_21_H_25_N_2_)_2_(OH)_2_]	*F*(000) = 816
*M**_r_* = 775.61	*D*_x_ = 1.355 Mg m^−^^3^
Monoclinic, *P*2_1_/*n*	Mo *K*α radiation, λ = 0.71073 Å
*a* = 12.8736 (4) Å	Cell parameters from 7335 reflections
*b* = 8.7682 (3) Å	θ = 2.3–28.3°
*c* = 17.4530 (5) Å	µ = 1.30 mm^−^^1^
β = 105.222 (2)°	*T* = 100 K
*V* = 1900.95 (10) Å^3^	Block, colorless
*Z* = 2	0.46 × 0.23 × 0.23 mm

### Data collection

**Table:**

Bruker APEX CCD diffractometer	3576 reflections with *I* > 2σ(*I*)
φ and ω scans	*R*_int_ = 0.016
Absorption correction: multi-scan (*SADABS*; Bruker, 2007)	θ_max_ = 26.0°, θ_min_ = 1.8°
*T*_min_ = 0.586, *T*_max_ = 0.754	*h* = −15→15
22627 measured reflections	*k* = −10→10
3730 independent reflections	*l* = −21→21

### Refinement

**Table:**

Refinement on *F*^2^	Primary atom site location: structure-invariant direct methods
Least-squares matrix: full	Secondary atom site location: difference Fourier map
*R*[*F*^2^ > 2σ(*F*^2^)] = 0.029	Hydrogen site location: mixed
*wR*(*F*^2^) = 0.079	H atoms treated by a mixture of independent and constrained refinement
*S* = 0.99	*w* = 1/[σ^2^(*F*_o_^2^) + (0.050*P*)^2^ + 1.390*P*] where *P* = (*F*_o_^2^ + 2*F*_c_^2^)/3
3730 reflections	(Δ/σ)_max_ = 0.002
230 parameters	Δρ_max_ = 1.06 e Å^−^^3^
0 restraints	Δρ_min_ = −0.25 e Å^−^^3^

### Fractional atomic coordinates and isotropic or equivalent isotropic displacement parameters (Å^2^)

**Table:**

	*x*	*y*	*z*	*U*_iso_*/*U*_eq_	
Zn1	0.95859 (2)	0.92266 (2)	0.92324 (2)	0.02197 (9)	
N1	1.01789 (11)	0.88907 (16)	0.83143 (8)	0.0201 (3)	
N2	0.81321 (11)	0.83790 (17)	0.87559 (8)	0.0228 (3)	
O1	0.96361 (10)	1.13369 (16)	0.96789 (7)	0.0259 (3)	
H1	0.9667 (18)	1.209 (3)	0.9400 (15)	0.038 (7)*	
C3	0.95099 (13)	0.87215 (19)	0.75919 (10)	0.0210 (3)	
C4	0.83960 (13)	0.8525 (2)	0.74383 (10)	0.0223 (3)	
H4	0.8007	0.8593	0.6896	0.027*	
C5	0.77700 (13)	0.8241 (2)	0.79754 (10)	0.0225 (3)	
C6	0.99662 (14)	0.8736 (2)	0.68752 (10)	0.0262 (4)	
H6A	1.0405	0.9654	0.6889	0.039*	
H6B	0.9375	0.8735	0.6389	0.039*	
H6C	1.0414	0.7829	0.6884	0.039*	
C7	0.66271 (13)	0.7706 (2)	0.76132 (10)	0.0267 (4)	
H7A	0.6537	0.6674	0.7802	0.040*	
H7B	0.6483	0.7698	0.7033	0.040*	
H7C	0.6123	0.8399	0.7770	0.040*	
C8	0.74730 (13)	0.7795 (2)	0.92370 (10)	0.0241 (4)	
C9	0.76109 (14)	0.6264 (2)	0.94808 (10)	0.0259 (4)	
C10	0.69362 (16)	0.5658 (2)	0.99114 (11)	0.0305 (4)	
H10	0.7011	0.4621	1.0074	0.037*	
C11	0.61560 (15)	0.6562 (3)	1.01037 (11)	0.0335 (4)	
H11	0.5684	0.6135	1.0383	0.040*	
C12	0.60651 (14)	0.8085 (3)	0.98893 (11)	0.0333 (4)	
H12	0.5541	0.8698	1.0037	0.040*	
C13	0.67270 (14)	0.8742 (2)	0.94604 (11)	0.0294 (4)	
C14	0.84837 (15)	0.5304 (2)	0.92859 (12)	0.0309 (4)	
H14A	0.8418	0.4248	0.9451	0.046*	
H14B	0.9191	0.5707	0.9568	0.046*	
H14C	0.8408	0.5334	0.8712	0.046*	
C15	0.66482 (18)	1.0416 (3)	0.92477 (14)	0.0394 (5)	
H15A	0.7365	1.0880	0.9413	0.059*	
H15B	0.6168	1.0925	0.9520	0.059*	
H15C	0.6360	1.0530	0.8672	0.059*	
C16	1.13079 (13)	0.8705 (2)	0.83836 (9)	0.0205 (3)	
C17	1.17176 (14)	0.7214 (2)	0.84038 (10)	0.0238 (3)	
C18	1.28096 (14)	0.7028 (2)	0.84487 (11)	0.0281 (4)	
H18	1.3101	0.6030	0.8463	0.034*	
C19	1.34732 (14)	0.8281 (2)	0.84731 (11)	0.0290 (4)	
H19	1.4212	0.8140	0.8494	0.035*	
C20	1.30597 (14)	0.9738 (2)	0.84663 (10)	0.0277 (4)	
H20	1.3522	1.0591	0.8488	0.033*	
C21	1.19747 (14)	0.9978 (2)	0.84288 (10)	0.0228 (3)	
C22	1.09984 (17)	0.5850 (2)	0.83697 (13)	0.0314 (4)	
H22A	1.0652	0.5902	0.8806	0.047*	
H22B	1.1429	0.4916	0.8419	0.047*	
H22C	1.0446	0.5843	0.7862	0.047*	
C23	1.15467 (15)	1.1575 (2)	0.84357 (11)	0.0285 (4)	
H23A	1.0852	1.1661	0.8038	0.043*	
H23B	1.2058	1.2301	0.8311	0.043*	
H23C	1.1452	1.1803	0.8963	0.043*	

### Atomic displacement parameters (Å^2^)

**Table:**

	*U*^11^	*U*^22^	*U*^33^	*U*^12^	*U*^13^	*U*^23^
Zn1	0.01621 (12)	0.03297 (14)	0.01712 (12)	0.00048 (7)	0.00511 (8)	−0.00413 (7)
N1	0.0170 (7)	0.0258 (7)	0.0179 (7)	0.0004 (5)	0.0054 (5)	−0.0020 (5)
N2	0.0172 (6)	0.0314 (8)	0.0201 (7)	0.0002 (6)	0.0056 (5)	0.0005 (6)
O1	0.0264 (6)	0.0323 (7)	0.0186 (6)	0.0012 (5)	0.0052 (5)	−0.0017 (5)
C3	0.0231 (8)	0.0214 (8)	0.0196 (8)	0.0010 (6)	0.0074 (6)	−0.0003 (6)
C4	0.0212 (8)	0.0272 (9)	0.0179 (8)	0.0000 (6)	0.0039 (6)	0.0015 (6)
C5	0.0190 (8)	0.0248 (8)	0.0226 (8)	0.0014 (6)	0.0037 (6)	0.0023 (7)
C6	0.0239 (8)	0.0366 (10)	0.0191 (8)	−0.0015 (7)	0.0073 (7)	−0.0002 (7)
C7	0.0204 (8)	0.0353 (10)	0.0227 (8)	−0.0037 (7)	0.0028 (7)	0.0038 (7)
C8	0.0164 (7)	0.0375 (10)	0.0177 (8)	−0.0011 (7)	0.0033 (6)	0.0005 (7)
C9	0.0215 (8)	0.0347 (9)	0.0201 (8)	−0.0020 (7)	0.0029 (7)	−0.0022 (7)
C10	0.0296 (10)	0.0388 (11)	0.0216 (9)	−0.0083 (8)	0.0040 (7)	0.0017 (7)
C11	0.0255 (9)	0.0542 (13)	0.0217 (9)	−0.0101 (9)	0.0081 (7)	−0.0004 (8)
C12	0.0205 (8)	0.0561 (13)	0.0243 (9)	0.0028 (8)	0.0078 (7)	−0.0023 (8)
C13	0.0208 (8)	0.0431 (11)	0.0242 (9)	0.0042 (8)	0.0057 (7)	0.0024 (8)
C14	0.0295 (9)	0.0311 (10)	0.0318 (10)	0.0015 (8)	0.0077 (8)	−0.0006 (8)
C15	0.0364 (11)	0.0441 (12)	0.0418 (12)	0.0127 (9)	0.0177 (9)	0.0040 (10)
C16	0.0187 (8)	0.0280 (9)	0.0157 (7)	0.0011 (6)	0.0062 (6)	−0.0011 (6)
C17	0.0251 (8)	0.0276 (9)	0.0204 (8)	0.0023 (7)	0.0087 (6)	−0.0009 (7)
C18	0.0274 (9)	0.0340 (10)	0.0248 (9)	0.0089 (7)	0.0102 (7)	0.0017 (7)
C19	0.0185 (8)	0.0461 (11)	0.0235 (9)	0.0032 (7)	0.0071 (7)	−0.0007 (8)
C20	0.0220 (8)	0.0392 (10)	0.0226 (9)	−0.0064 (7)	0.0072 (7)	−0.0025 (8)
C21	0.0243 (8)	0.0281 (9)	0.0167 (8)	−0.0002 (7)	0.0066 (6)	−0.0006 (6)
C22	0.0341 (10)	0.0249 (9)	0.0386 (11)	0.0014 (7)	0.0154 (9)	−0.0015 (7)
C23	0.0315 (9)	0.0258 (9)	0.0296 (9)	−0.0016 (7)	0.0104 (7)	−0.0007 (7)

### Geometric parameters (Å, º)

**Table:**

Zn1—O1^i^	1.9643 (13)	C11—H11	0.9500
Zn1—N1	1.9696 (14)	C12—C13	1.397 (3)
Zn1—N2	1.9823 (14)	C12—H12	0.9500
Zn1—O1	2.0022 (14)	C13—C15	1.511 (3)
N1—C3	1.335 (2)	C14—H14A	0.9800
N1—C16	1.436 (2)	C14—H14B	0.9800
N2—C5	1.324 (2)	C14—H14C	0.9800
N2—C8	1.436 (2)	C15—H15A	0.9800
O1—H1	0.83 (3)	C15—H15B	0.9800
C3—C4	1.398 (2)	C15—H15C	0.9800
C3—C6	1.515 (2)	C16—C21	1.398 (2)
C4—C5	1.410 (2)	C16—C17	1.407 (2)
C4—H4	0.9500	C17—C18	1.397 (2)
C5—C7	1.515 (2)	C17—C22	1.504 (3)
C6—H6A	0.9800	C18—C19	1.386 (3)
C6—H6B	0.9800	C18—H18	0.9500
C6—H6C	0.9800	C19—C20	1.383 (3)
C7—H7A	0.9800	C19—H19	0.9500
C7—H7B	0.9800	C20—C21	1.397 (2)
C7—H7C	0.9800	C20—H20	0.9500
C8—C13	1.400 (3)	C21—C23	1.506 (3)
C8—C9	1.406 (3)	C22—H22A	0.9800
C9—C10	1.394 (3)	C22—H22B	0.9800
C9—C14	1.512 (3)	C22—H22C	0.9800
C10—C11	1.388 (3)	C23—H23A	0.9800
C10—H10	0.9500	C23—H23B	0.9800
C11—C12	1.383 (3)	C23—H23C	0.9800
			
O1^i^—Zn1—N1	122.77 (6)	C11—C12—C13	121.50 (18)
O1^i^—Zn1—N2	119.80 (6)	C11—C12—H12	119.2
N1—Zn1—N2	97.33 (6)	C13—C12—H12	119.2
O1^i^—Zn1—O1	84.25 (6)	C12—C13—C8	117.67 (19)
N1—Zn1—O1	118.36 (6)	C12—C13—C15	121.39 (18)
N2—Zn1—O1	116.11 (6)	C8—C13—C15	120.94 (17)
C3—N1—C16	117.04 (14)	C9—C14—H14A	109.5
C3—N1—Zn1	119.54 (11)	C9—C14—H14B	109.5
C16—N1—Zn1	123.26 (11)	H14A—C14—H14B	109.5
C5—N2—C8	117.81 (14)	C9—C14—H14C	109.5
C5—N2—Zn1	120.29 (11)	H14A—C14—H14C	109.5
C8—N2—Zn1	121.74 (11)	H14B—C14—H14C	109.5
Zn1^i^—O1—Zn1	95.75 (6)	C13—C15—H15A	109.5
Zn1^i^—O1—H1	132.7 (17)	C13—C15—H15B	109.5
Zn1—O1—H1	120.8 (17)	H15A—C15—H15B	109.5
N1—C3—C4	124.70 (15)	C13—C15—H15C	109.5
N1—C3—C6	118.99 (15)	H15A—C15—H15C	109.5
C4—C3—C6	116.30 (15)	H15B—C15—H15C	109.5
C3—C4—C5	129.11 (15)	C21—C16—C17	121.33 (15)
C3—C4—H4	115.4	C21—C16—N1	120.49 (15)
C5—C4—H4	115.4	C17—C16—N1	118.17 (15)
N2—C5—C4	124.02 (15)	C18—C17—C16	118.38 (16)
N2—C5—C7	119.89 (15)	C18—C17—C22	120.61 (17)
C4—C5—C7	116.07 (15)	C16—C17—C22	121.01 (15)
C3—C6—H6A	109.5	C19—C18—C17	120.81 (17)
C3—C6—H6B	109.5	C19—C18—H18	119.6
H6A—C6—H6B	109.5	C17—C18—H18	119.6
C3—C6—H6C	109.5	C20—C19—C18	119.98 (16)
H6A—C6—H6C	109.5	C20—C19—H19	120.0
H6B—C6—H6C	109.5	C18—C19—H19	120.0
C5—C7—H7A	109.5	C19—C20—C21	121.15 (18)
C5—C7—H7B	109.5	C19—C20—H20	119.4
H7A—C7—H7B	109.5	C21—C20—H20	119.4
C5—C7—H7C	109.5	C20—C21—C16	118.32 (17)
H7A—C7—H7C	109.5	C20—C21—C23	120.18 (17)
H7B—C7—H7C	109.5	C16—C21—C23	121.50 (16)
C13—C8—C9	121.56 (16)	C17—C22—H22A	109.5
C13—C8—N2	120.37 (17)	C17—C22—H22B	109.5
C9—C8—N2	118.06 (15)	H22A—C22—H22B	109.5
C10—C9—C8	118.67 (17)	C17—C22—H22C	109.5
C10—C9—C14	120.93 (18)	H22A—C22—H22C	109.5
C8—C9—C14	120.39 (16)	H22B—C22—H22C	109.5
C11—C10—C9	120.38 (19)	C21—C23—H23A	109.5
C11—C10—H10	119.8	C21—C23—H23B	109.5
C9—C10—H10	119.8	H23A—C23—H23B	109.5
C12—C11—C10	120.06 (17)	C21—C23—H23C	109.5
C12—C11—H11	120.0	H23A—C23—H23C	109.5
C10—C11—H11	120.0	H23B—C23—H23C	109.5
			
C16—N1—C3—C4	165.14 (16)	C11—C12—C13—C8	1.5 (3)
Zn1—N1—C3—C4	−10.5 (2)	C11—C12—C13—C15	−178.18 (19)
C16—N1—C3—C6	−14.8 (2)	C9—C8—C13—C12	−4.5 (3)
Zn1—N1—C3—C6	169.60 (12)	N2—C8—C13—C12	176.31 (16)
N1—C3—C4—C5	−10.3 (3)	C9—C8—C13—C15	175.20 (17)
C6—C3—C4—C5	169.62 (18)	N2—C8—C13—C15	−4.0 (3)
C8—N2—C5—C4	−169.09 (16)	C3—N1—C16—C21	100.95 (19)
Zn1—N2—C5—C4	6.4 (2)	Zn1—N1—C16—C21	−83.61 (18)
C8—N2—C5—C7	9.1 (2)	C3—N1—C16—C17	−79.0 (2)
Zn1—N2—C5—C7	−175.34 (12)	Zn1—N1—C16—C17	96.43 (16)
C3—C4—C5—N2	12.6 (3)	C21—C16—C17—C18	−1.7 (2)
C3—C4—C5—C7	−165.64 (18)	N1—C16—C17—C18	178.23 (15)
C5—N2—C8—C13	−91.7 (2)	C21—C16—C17—C22	178.88 (17)
Zn1—N2—C8—C13	92.82 (17)	N1—C16—C17—C22	−1.2 (2)
C5—N2—C8—C9	89.03 (19)	C16—C17—C18—C19	0.0 (3)
Zn1—N2—C8—C9	−86.44 (18)	C22—C17—C18—C19	179.42 (17)
C13—C8—C9—C10	4.2 (3)	C17—C18—C19—C20	1.1 (3)
N2—C8—C9—C10	−176.53 (15)	C18—C19—C20—C21	−0.5 (3)
C13—C8—C9—C14	−175.09 (16)	C19—C20—C21—C16	−1.1 (3)
N2—C8—C9—C14	4.2 (2)	C19—C20—C21—C23	179.00 (16)
C8—C9—C10—C11	−0.9 (3)	C17—C16—C21—C20	2.3 (2)
C14—C9—C10—C11	178.37 (17)	N1—C16—C21—C20	−177.69 (15)
C9—C10—C11—C12	−2.0 (3)	C17—C16—C21—C23	−177.87 (16)
C10—C11—C12—C13	1.7 (3)	N1—C16—C21—C23	2.2 (2)

Symmetry code: (i) −*x*+2, −*y*+2, −*z*+2.
