# Supplementary material for: Crystal structure of di-μ-hydroxido-bis{[N,N′-bis­(2,6-di­methyl­phen­yl)pentane-2,4-diiminato(1–)]zinc}
Source: Acta Crystallogr Sect E Struct Rep Online. 2014 Aug 6;70(Pt 9):m320–1. doi: 10.1107/S160053681401736X (PMC4186180; doi:10.1107/S160053681401736X)
Supplement: Supplementary file 2 [file e-70-0m320-Isup2.docx]

1. Comments

As a part of our effort to investigate the activation of CO_2_ by functional/structural models of carbonic anhydrase, a dinuclear zinc(II) complex with a Zn_2_(OH)_2_ core structure supported by β-diiminate ligands was obtained and studied. The zinc(II) dimer is centrosymmetric and the two zinc centers are connected by two μ-hydroxide bridging ligands. Each zinc center possesses a distorted tetrahedral geometry (*τ*_4_ = 0.83, Yang & Houser 2007) with two nitrogen donors from the β-diiminate ligands and two oxygen donors from the two OH^-^ groups. The zinc•••zinc distance is about 2.942 Å (Fig. 1). Similar dinuclear zinc(II) complexes with β-diiminate ligands decorated with different functional groups have also been studied for copolymerization of CO_2_ and epoxide (Gondzik & Schulz (2014); Schulz & Spielmann (2011); Chisholm and Gallucci (2002); Cheng & Moore (2001)).

2. Experimental

The complex was synthesized from the reaction of water with [Zn(Et)(*N*,*N*'-bis(2,6-dimethylphenyl)pentane-2,4-diiminato)] complex in Et_2_O. The solvent was removed under vacuum after the reaction and white powder was collected. Recrystallization of the white powder in saturated Et_2_O in -20°C led to the formation of colorless crystals suitable for X-ray crystallography analysis.

3. Refinement

The monoclinic space group *P*2_1_/*n* was determined by systematic absences and statistical tests and verified by subsequent refinement. The structure was solved by direct methods and refined by full-matrix least-squares methods on F^2^ (3). The positions of hydrogens bonded to carbons were initially determined by geometry and refined by a riding model. The hydrogen bonded to the oxygen was located on a difference map, and its position was refined independently. Non-hydrogen atoms were refined with anisotropic displacement parameters. Hydrogen atom displacement parameters were set to 1.2 (1.5 for methyl) times the isotropic equivalent displacement parameters of the bonded atoms. A total of 230 parameters were refined against 3730 data to give wR(F*2*) = 0.0789 and S = 0.994 for weights of w = 1/[σ^2^ (*F*^2^) + (0.0500 P)^2^ + 1.3900 P], where P = [*F*_o_2 + 2*F*_c_^2^] / 3. The final R(*F*) was 0.0285 for the 3576 observed, [*F* > 4*σ*(*F*)], data. The largest shift/s.u. was 0.002 in the final refinement cycle. The final difference map had maxima and minima of 1.064 and -0.248 e/Å^3^, respectively.


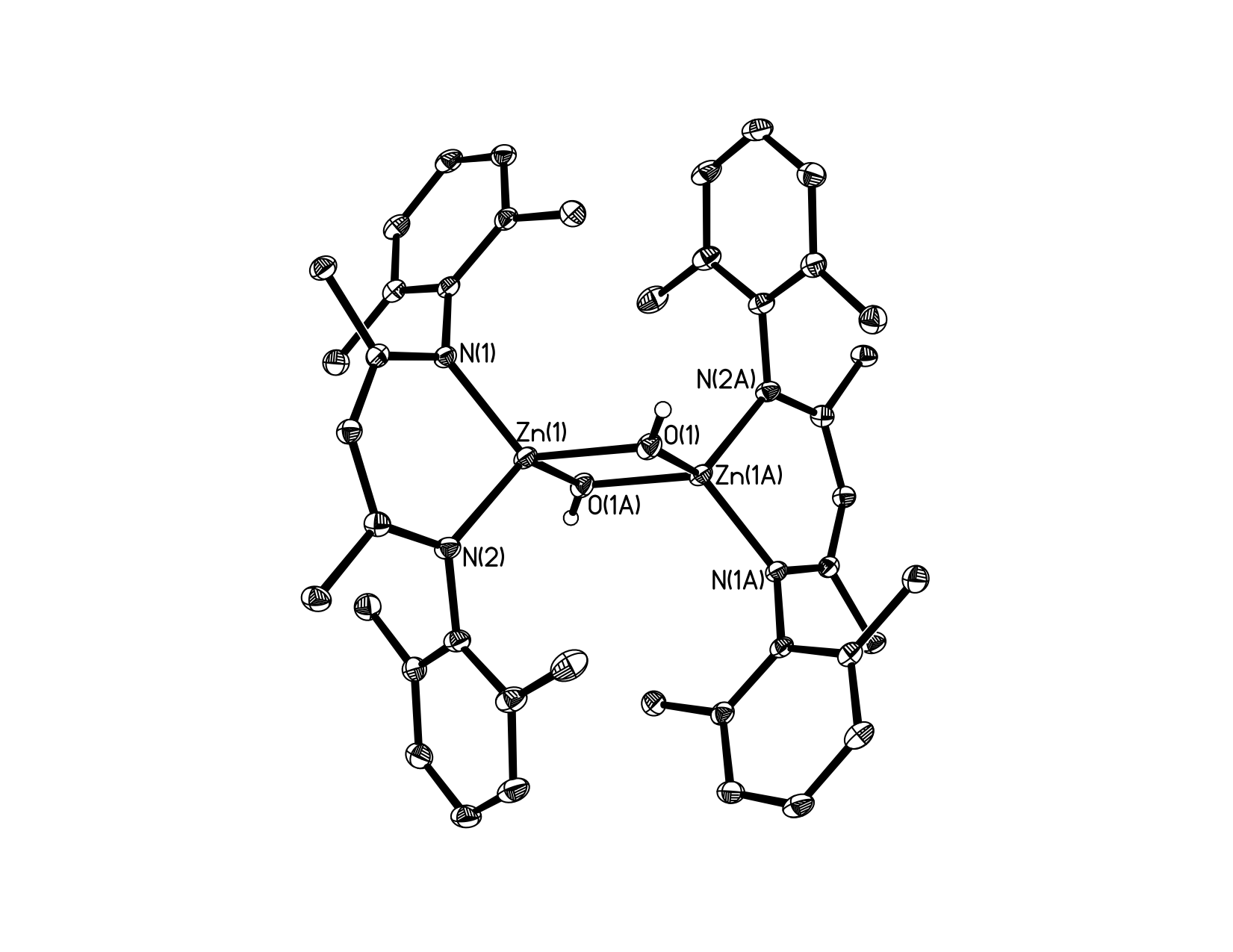


Figure 1

The crystal structure of the title complex. Anisotropic displacement ellipsoids were drawn at the 30% probability level. Hydrogen atoms except for the two on oxygen donors have been omitted for clarity. Symmetry code: #1 -x+2, -y+2, -z+2
