# Supplementary figures and images for: Crystal structure of di-μ-hydroxido-bis{[N,N′-bis­(2,6-di­methyl­phen­yl)pentane-2,4-diiminato(1–)]zinc}
Source: Acta Crystallogr Sect E Struct Rep Online. 2014 Aug 6;70(Pt 9):m320–1. doi: 10.1107/S160053681401736X (PMC4186180; doi:10.1107/S160053681401736X)

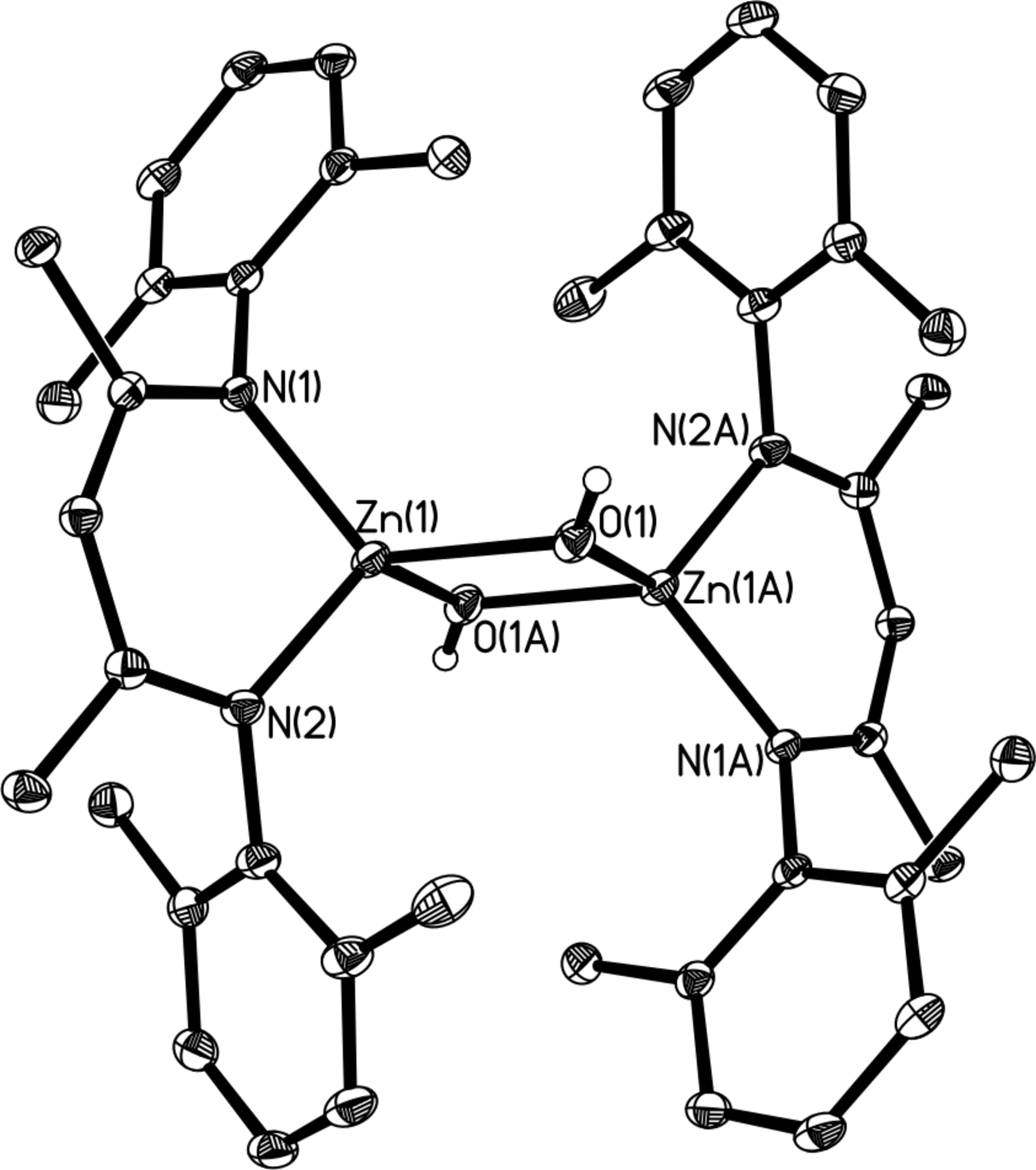

Supplement: Supplementary file 5 [file e-70-0m320-fig1.tif]
